# Role of Cardiorespiratory Fitness, Aerobic, Exercise and Sports Participation in Female Cognition: A Scoping Review: Sports, Fitness, and Cognition

**DOI:** 10.1186/s40798-024-00776-8

**Published:** 2024-09-27

**Authors:** Vinicius Muller Reis Weber, Marcos Roberto Queiroga, Jessica L. Puranda, Kevin Semeniuk, Meaghan Lindsay Macdonald, Diego Bessa Dantas, Danilo Fernandes da Silva, Kristi Bree Adamo

**Affiliations:** 1 Laboratory of Experimental and Applied Physiology to Physical Activity, UNICENTRO, Street Alameda Elio Antonio Dalla Vecchia, 838, Vila Carli, Guarapuava, Paraná 85040-167 Brazil; 2 Associated Graduate Program in Physical Education UEM/UEL, Londrina, Brazil; 3 Faculty of Health Sciences, School of Human Kinetics, University of Ottawa, Ottawa, ON Canada; 4 Sports Studies Department, Bishop’s University, Sherbrooke, QC Canada

**Keywords:** Executive function, Cognition, Sports, Fitness, Aerobic exercise, Female

## Abstract

**Background:**

The impact of cardiorespiratory fitness (CRF) on cognition is thought to be mediated by brain-derived neurotrophic factor. Aerobic exercise can increase CRF through various activities, including sports participation. The relationship between these factors in females has yet to be elucidated.

**Objective:**

This review aims to map the current literature on the effects of aerobic exercise, sports participation, and CRF in healthy adult females, with sub-topics of pregnancy and menstrual cycle periodicity.

**Methods:**

A scoping review of the literature was conducted following PRISMA guidelines and the PCC mnemonic (population, concept, and context). The following five databases were screened: CINAHL, Medline, Web of Science, SPORTDiscus, and Scopus. Eligible articles included healthy adult females, investigated aerobic exercise, sports participation or CRF, and linked outcomes to cognition. Data from included manuscripts was extracted and analyzed. Two sub-population groupings (pregnant individuals and menstrual cycle) were established to further aid the interpretation of the findings.

**Results:**

Of the 300 titles and abstracts screened, 74 were eligible for full-text screening, and 28 were included in the scoping review. Of the 28 included, 14 did not control for or report on menstrual cycle phase or sex hormones.

**Conclusion:**

This scoping review found an inverse ‘U’ relationship between aerobic exercise and cognition, demonstrating an optimal dose of aerobic exercise to benefit cognitive functions. As estrogen may impact the relationship between CRF and neural growth factors, more research is needed on this pathway, independent of the menstrual cycle, to determine potential beneficial effects. It is currently unknown whether sports participation can independently impact cognition.

## Key Points

- Regular sports participation enhances executive functions, brain activation, and BDNF levels.
- Both acute and chronic aerobic exercise improve cognition, but excessive exercise can reduce BDNF and impair cognitive performance. Highlighting the possible inverted “U” theory.
- Hormonal changes during the menstrual cycle and pregnancy affect cognitive functions and BDNF levels, with exercise offering possible protective effects.

## Background

Low levels of cardiorespiratory fitness (CRF) have detrimental effects on population health and have been exacerbated with the rise in sedentary behavior during the COVID-19 pandemic [1–3]. Low levels of CRF are associated with obesity [4], metabolic syndrome [5], poor bone health [6], and anxiety and depression [1, 7, 8]. Pertaining to mental health, CRF is directly related to the expression of the molecule brain-derived neurotrophic factor (BDNF) [9–11]; where low levels of this protein are associated with major depression [11, 12].

BDNF contributes on neuroprotection, neurogenesis, memory consolidation, brain excitability, and neural connectivity [13–15]. Moreover, the effects of CRF on cognition seem to be mediated by BDNF levels [16, 17]. When examining aspects of cognition, the executive function appears to be most influenced by CRF [18–20]. Executive function refers to series of cognitive processes responsible for action plans and decision making; its features include domains such as inhibitory control (i.e., inhibit distraction stimulus that can lead to a wrong action), working memory, and mental flexibility [21, 22], and are essential for daily tasks (e.g., managing money; managing home) [23] as well as academic achievement [24].

One way to improve CRF is by engaging in habitual aerobic exercise [25]. A category of aerobic exercise is sports participation. Individuals exposed to sports training that involve physical exertion, especially aerobic exercises, usually exhibit increased CRF [26, 27]. More than that, sports participation can be divided in open (i.e., basketball, soccer, hockey) and closed skills sports (i.e., swimming, running). Open skill sports are those which players are required to consistently react and adapt to an unpredictable environment. Whereas closed skill sports are defined by sports with a stable environment, during which players have a predetermined movement pattern [28].

More than being physically demanding, sports participation can also require the high utilization of different cognitive aspects (e.g., attention, inhibitory control, cognitive flexibility) [29]. To significantly improve sports performance it is necessary to integrate these cognitive functions and enhance the top-down processing (i.e., utilize past experiences to guide an activity or reaction) [30, 31]. During sports participation, players must be attentive to different environmental changes, and adapt to complex and quickly changing conditions [22]. However, there is a lack of literature examining the relationship between sport-based and non-sport physical activity and cognition [32]. What remains to be clarified or determined is whether or not engaging in sports participation offers cognitive advantages over regular engagement in physical activity, since executive functions are correlated to health related variables (i.e. physical fitness) [29].

During aerobic exercise, skeletal muscle contractions upregulate BDNF release [33, 34], which can result in BDNF being stored in blood platelets [35]. Additionally, blood platelets promote homeostasis by repairing vessels, promoting clotting, and increasing inflammatory responses [36]. There is an effect of CRF on platelet activity owing to muscular and vascular adaptations to habitual physical activity/training [37, 38]. Moreover, in animals models, circulating BDNF can cross the blood–brain barrier, and peripheral BDNF (e.g. serum BDNF) is strongly related to the amount of BDNF in the brain [39]. Another protein related to cardiorespiratory fitness (CRF), muscle contraction, and brain health is vascular endothelial growth factor (VEGF). VEGF plays a crucial role in promoting angiogenesis. Increasing VEGF dynamics with exercise [40], may enhance cerebral blood flow, which is essential for supporting neurogenesis [41].

Exposure to aerobic exercise results in hormonal changes. Strenuous activities can increase cortisol levels, catecholamine release, and decrease energy resources. These alterations in whole body homeostasis can overstimulate cognitive functions. For example, during a strenuous exercise, the body increases catecholamine levels and glucose consumption [42]; these patterns can result in neural noise due to high levels of catecholamines [9, 43] or decreasing brain excitability by decreasing energetic resources during/after intense activities [44, 45]. Therefore, determining what the optimal dose of aerobic exercise for improvements in cognitive function is necessary.

Biological sex is an important consideration when examining CRF as well as aspects of cognition as there are known between-sex differences. These differences occur mainly because of sex hormones since estrogen is strongly related to BDNF [46]. Also, engaging in exercise may have more significant impacts on cognition in females [47]. The promotion of cognitive health with exercise during adulthood may be protective against the deleterious effects of age, reduction of sex hormones and chemical dysregulations on cognitive functions [9, 29, 47]. During adulthood, menstruation and pregnancy are uniquely female experiences that may play a role in cognitive functions and hormone release. All these aspects must be addressed to elucidate the possible female-centric impact of CRF/aerobic activity on cognitive-related outcomes. Cognitive-related outcomes are measures/effects related to the function of brain and mental process which encompass cognitive function, growth factors and other biomarkers that may influence cognitive function and brain imaging.

The available literature predominantly focuses on males or mixed samples, creating a gap in research regarding the effects of CRF, aerobic exercise, and cognition specifically in the female population. It is important to highlight that males and females experience significantly different impacts on cognition [32, 47] and CRF [48] through their lifespan. These differences complicate the application of findings across the sexes. Therefore, the purpose of this study is to map the research done and identify the gaps related to the effects of aerobic exercise, sports participation, and cardiorespiratory fitness on cognition in healthy adult females, with sub-topics related to menstrual cycle periodicity and pregnancy. Considering the wide scope of our topic and the limited existing literature, a scoping review is one of the most suitable methods for identifying and analyzing gaps in the literature [49].

## Main Text

### Methods

#### Inclusion and Exclusion Criteria

A scoping review was conducted, using the PCC mnemonic (population, concept, and context) to develop the research question [49], to elucidate the state of the literature on the relationship between aerobic exercise, sports participation, cardiorespiratory fitness, and cognition in a healthy female population. This study followed the recommendations of PRISMA-ScR checklist [50].

Eligible articles included the following aspects: (1) healthy adult female individuals; (2) investigated any aerobic exercise, sports participation, or cardiorespiratory fitness; and (3) the outcomes were linked to cognition. Articles that included male participants, pathology, animals, elderly, and when the objective of the study was to verify the effects of illicit or admissible/legal drug use on cognition, were excluded.

### Search Strategy

The initial search was performed on October 30th, 2022, and a second search, to refine the articles was performed on September 13th, 2023. The following databases were screened: Cinahl, Medline, Web of Science, Sport Discus, and Scopus. The keywords, MeSH terms, and Boolean operators used to facilitate the search are detailed in Table 1. For this review no restriction for language and date was utilized. The articles eligible for the title/abstract screening were transferred to Covidence, and duplicate articles were automatically removed. Two reviewers independently screened the manuscripts by title and abstract; if any conflict was identified, a third review was consulted. The reference lists of included articles were checked for potentially relevant studies that met our inclusion criteria.

**Table 1:** Search strategy for Medline

Key term	Search strategy	Retrieved articles
Aerobic Exercise	("Sports" OR "Sport" OR “Athletes” OR “Physical Fitness” OR “Cardiorespiratory Fitness” OR “Aerobic Exercise”)	370,317
Cognitive outcomes	("Brain-Derived Neurotrophic Factor" OR "Nerve Growth Factors" OR "Vascular Endothelial Growth Factors" OR "Executive Function" OR "Inhibitory Control" OR “Stroop Task” OR "Working Memory" OR "receptor TrkB" OR “Cognitive Flexibility”)	135,124
Females	("Woman" OR "Female" OR “Pregnant women” OR “Menstrual Cycle” OR “Reproductive Health”)	9,913,245
	*Above searches combined with AND*	1456
AND NOT	(“Male” OR “Elderly” OR “Aged” OR “Older” OR “Children” OR “Child” OR “Adolescents” OR “Concussion” OR “Dementia” OR “Alzheimer”)	97

### Data Extraction and Synthesis

After reviewing the full text, information was extracted from the included manuscripts utilizing the Covidence software, and the following information was summarized: author, country, study design, study population, outcomes, and summary of main findings. To address the research question, the reviewers grouped the findings by the predictor variables (i.e., aerobic exercise, sports participation, and cardiorespiratory fitness) to summarize the main findings. The two reviewers also analyzed the relationship between aerobic exercise, sports participation, cardiorespiratory fitness, and cognition in two female sub-populations (i.e., pregnant individuals, individuals with menstrual cycle variations). The sub-population groupings were used to aid in the interpretation of findings to answer the research question.

## Results

**Fig. 1:**
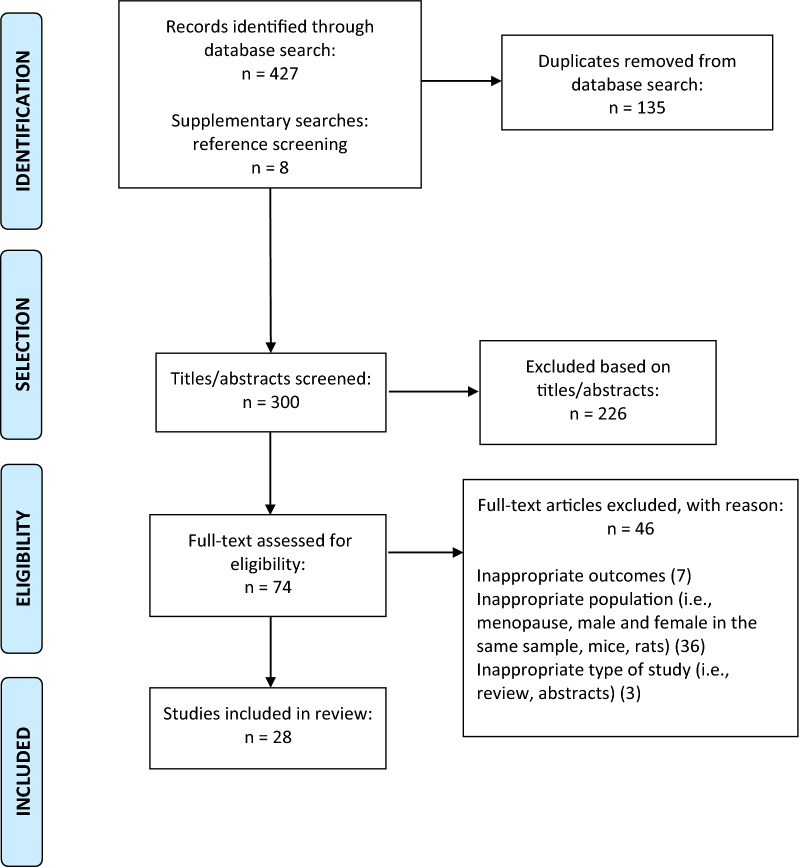
PRISMA flow diagram

The database search and references screening identified a total of 435 articles (427 from database search and 8 from references screening), of which 135 were duplicates. A total of 300 titles and abstracts were screened, and 74 studies were eligible for full-text screening. Of the remaining articles identified, 28 studies were included in the scoping review. All stages of the screening process are presented in Fig. 1.

Of the 28 articles included, 14 did not control for or report information on the menstrual cycle phase or sexual hormones of the participants included. Additional information and a summary of the findings from individual studies can be found in Tables 2 and 3. Table 2 summarizes the association between aerobic exercise, sports participation, cardiorespiratory fitness, and cognition in a healthy female population. Table 3 includes a summary of the effect of pregnancy and menstrual cycle periodicity on the relationship between aerobic exercise, sports participation, cardiorespiratory fitness, and cognition in a healthy female population.

**Table 2:** - Characteristics of studies analyzing the effects of cardiorespiratory fitness, aerobic exercise and sports on cognition

Author (year)/country	Study type	Total sample	Context	Cognitive outcome	Main findings
*Cardiorespiratory fitness and cognitive outcomes in healthy female individuals*					
Scott et al. [20] USA	Cross-sectional	120	Cardiorespiratory fitness	Executive function	VO_2_ peak is positively associates to attention (*P* < 0.01), shifting (*P* < 0.01) and working memory (*P* < 0.01)
Li et al. [52] China	Cross-sectional	24	Cardiorespiratory fitness	Executive function and Brain images	High-fit group showed greater brain activation in the anterior cortex and has higher accuracy (*t(11)* = *2.315; p* = *0.03)*
Schmalhofer., [50] Germany	Cross-sectional	822	Cardiorespiratory fitness	Serum BDNF (pg/ml)	VO_2_ peak is positively associated with BDNF (β: 2.35; CI: 1.17 – 3.52)
Cui et al. [51] China	Cross-sectional	115	Cardiorespiratory fitness and acute moderate exercise (30 min)	Inhibitory control and Brain images	Acute exercise decreases the RT of low-fit group High-fit group showed greater brain activation than the low-fit group in the post-rest imaging, mainly in the anterior cortex
*Aerobic exercise and cognitive outcomes in healthy female individuals*					
Nofuji et al. [53] Japan	Cross-sectional	8 controls 8 physically active	Acute low, moderate, and maximum aerobic exercise (30 min)	Serum BDNF (pg/ml)	BDNF increased immediately after maximal and moderate exercise for the sedentary and active groups (p < 0.01) BDNF decreased for active group after 30 (-15%) and 60 min (-25%) of maximum exercise
Schmidt-kassow et al. [54] Germany	Cross-sectional	20	Acute low-intensity and high-intensity aerobic exercise (30 min)	Serum BDNF (pg/ml)	Increase of BDNF during high intensity exercise Exercise: 31,392.1 baseline: 30,221.5
Li et al. [57] China	Cross-sectional	15	Acute moderate-intensity aerobic exercise (30 min)	fMRI, working memory	Acute exercise activates prefrontal cortex but not changes working memory performance (*P* > *0.05)*
Lieberman et al. [59] USA	Longitudinal	109	Basic combat training (BCT)/Military (10 weeks)	Reaction time, Working memory	Increase in RT after BCT *d*: 0.47; P = 0.016
Hwang et al. [55] USA	Cross-sectional	14	Acute high-intensity aerobic exercise (20 min)	Serum BDNF (pg/ml)	Increase of BDNF immediately after high intensity exercise and decreases during recovery Exercise: 23,492 Baseline: 20,989 Recovery: 19,919
Lowe et al. [58] Canada	Cross-sectional	51	Acute moderate-intensity aerobic exercise (20 min)	Inhibitory control	Better performance after acute exercise (F(1,49) = 13.729, *P* = 0.001)
Jürimäe et al. [56] Estonia	Cross-sectional	15	Acute sub-maximal exercise (1 h)	Plasma VEGF (pg/ml)	VEGF significant increases immediately after post-exercise compared to pre-exercise CV: 1.70 ES: 0.19
Rentería et al. [60] USA	Randomized controlled trial	17	Short-term HIIT program (4 weeks) and GXT	Serum BDNF (pg/ml)	HIIT increases BDNF before GXT compared to control (P < 0.05) Decreases of BDNF after GXT for HIIT group are higher (P < 0.001)
Luo et al. [61] China	Randomized controlled trial	70	HICT program (12 weeks)	Inhibitory control	Faster incongruent RT after HICT (*d*:0.38; P = 0.047)
*Aerobic exhaustion exercise and cognitive outcomes in healthy female individuals*					
Bue-estes et al. [62] USA	Cross-sectional	26	Maximal aerobic exercise	Reaction time, Working memory, Visual spatial Memory	Working memory significantly lower when intensity was up to 50% of VO_2_ max Higher working memory after recovery time (after 30 min)
García-Suárez et al. [63] Mexico	Cross-sectional	17	Acute effect of GXT and HIIT	Serum BDNF (ng/ml), cortisol (μg/dl)	HIIT increases BDNF post-exercise *d:* 0.17 GXT decreases BDNF post-exercise *d:* -0.26 The ratio of cortisol and BDNF increases after exertion
Conkright et al. [64] USA	Cross-sectional	15	Physical exertion (TMT) / Military (3 days)	Plasma BDNF (pg/ml) and Serum cortisol (μg/dl)	TMT did not change BDNF levels TMT increases cortisol (p: < 0.05)
Armstrong et al. [65] UK	Cross-sectional	10	Physical exertion (3-h loaded march)/ Military	Inhibitory control, working memory, military tasks	High and Very-high loaded reduced working memory Inhibitory control was reduced in high loaded
*Sports practice and cognitive outcomes in healthy female individuals*					
Schor et al. [66] Brazil	Cross-sectional	15 professional judo fighters	Training session and GXT	Plasma BDNF (pg/ml)	BDNF increases after both tests Delta BDNF was higher during training session (P = 0.003)
Shi et al. [67] China	Cross-sectional	20 soccer athletes 15 aerobic athletes 15 controls	sports practice	Inhibitory control and fMRI	The soccer and aerobic groups presented with lower RT than control. However, the soccer group presented with lower inhibitory RT The soccer group presented more activation of basal nuclei than aerobic groups
Pradas et al. [68] Spain	Cross-sectional	14 padel athletes	Competition	Blood BDNF ng/ml	Padel competition increased BDNF (pre: 1531.12 × post: 1769.56; *d*:1.527; p < *0.05*)
Yu et al. [69] China	Cross-sectional	38 ice hockey	Skill level	fNIRS and executive function	The accuracy and reaction time is better for elite players (*p* = 0.001). Also, the elite group had higher activation of prefrontal (*p* = 0.026) and frontal cortex (*p* = 0.03)

BDNF, brain derived neurotrophic factor; fMRI, functional magnetic resonance imaging; fNIRS, functional near-infrared spectroscopy; GXT, graded exercise texting; HICT, high-intensity circuit training; HIIT, high-intensity interval training; N.S., not significant; RT, reaction time; VEGF, vascular endothelial growth factor; VO_2_ max, maximum oxygen consumption; VO_2_ peak, peak oxygen consumption

**Table 3:** - Characteristics of studies analyzing the effects of menstrual cycle and pregnancy on the relationship between cardiorespiratory fitness, aerobic exercise, and sports with cognition

Author (year) country	Study type	Total Sample	Context	Cognitive outcome	Main findings
*Relationship between cardiorespiratory fitness, aerobic exercise, cognitive outcomes, and menstrual cycle periodicity*					
Melin et al. [70] Denmark and Sweden	Cross-sectional	16 EUM 14 AM	Acute maximal aerobic exercise (2 bouts)	BDNF (μg/L), Cortisol (nmol/L)	Acute exercise increased cortisol (+ 98.6) and BDNF (+ 96.5) only in AM group
Nose et al. [71] Japan	Cross-sectional	132 EUM 63 AM	Elite Athletes	Serum BDNF (ng/ml), serum estradiol (pg/ml)	AM presented lower BDNF than EUM (median: 22.9 × 25.2) A significant relationship between BDNF and estradiol (r: 0.209)
Dirk et al. [72] Canada	Longitudinal	15 EUM	Acute aerobic exercise during Follicular and Luteal Phase (20 min)	Inhibitory control RT	Acute exercise decreased RT for both menstrual phases (*P:*0.003) Menstrual phase did not impact RT
Poli et al. [73] Brazil	Longitudinal	14 EUM	Acute HIIE during Follicular and Luteal Phase (20 min)	Inhibitory control, serum BDNF (pg/ml)	BDNF increased after HIIE for both conditions (LUT: + 8.22; FOL: + 7.29) VO_2_max is related to ΔBDNF after HIIE during follicular phase (r: -0.539)
*Relationship between cardiorespiratory fitness, aerobic exercise, cognitive outcomes, and pregnancy*					
Rojas-Vega et al. [74] Germany	Cross-sectional	20 3rd trimester	Sub maximum GXT (150 bpm) pre- and post-partum	Serum BDNF (ng/dl), VEGF (pg/ml), cortisol (μg/dl)	BDNF increased during sub-maximum exercise for pregnant individuals (p = 0.048) BDNF is higher and cortisol is lower after childbirth (p < 0.001)
LeMoyne et al. [76] Canada	Cross-sectional	52 pregnant (1st trimester 15, 2nd trimester:18; 3rd trimester: 10) 15 control	Cardiorespiratory fitness	Inhibitory control	Inhibitory control is negatively impacted by pregnancy (F: 2.86; p = 0.04). VO_2_max changes during the pregnancy (F:4.61; p = 0.006)
Ferrari et al. [75] Germany	Longitudinal	19 intervention 15 control	Moderate combined exercise from 14th week to 30th week of gestation	Serum BDNF (pg/ml)	BDNF is higher in the exercise group compared to the control group (control: 3371.2 × INT: 6540.7; p < 0.001)

EUM, eumenorrheic; AM, amenorrheic; BDNF, brain derived neurotrophic factor; GXT—grade exercise testing; FOL, Follicular phase; HIIE, high intensity interval exercise; LUT, luteal phase; RT, reaction time; VEGF, vascular endothelial growth factor; VO_2_ max, maximum oxygen consumption

### Cardiorespiratory Fitness and Cognitive-Related Outcomes

A limited number of studies (n = 4) were found that examined the effects of cardiorespiratory fitness on markers of cognition [20, 51–53]. All assessed cardiorespiratory fitness by maximal oxygen consumption (VO_2max_), during a graded exercise test, and none of these studies controlled for menstrual phase or sexual hormones. In one study [51], a positive relationship was shown between CRF and serum BDNF. Also, females categorized as the high-fit group (VO_2max_ in the 50th percentile or above) had higher activation of the anterior cortex during executive function tasks and better accuracy during rest [52, 53]. Similar to the other findings, CRF was related to different aspects of executive function, namely working memory and shifting attention [20].

### Aerobic Exercise and Cognitive-Related Outcomes

The impact of aerobic exercise on cognition was examined in nine articles, of which six assessed the effect of acute aerobic exercise [54–59] and three assessed the effect of chronic aerobic intervention [60–62].

The results from four studies showed that an acute (single) bout of aerobic exercise (> 60% of VO_2peak_ intensity) can increase serum BDNF, vascular endothelial growth factor (VEGF) [54–56] and increase inhibitory control (50% of maximum heart rate) [59]. No effects of light-intensity aerobic activity were found for neural markers. Also, moderate exercise (60–70% of maximum heart rate) appears to modulate brain areas responsible for executive functions, for example, activating the prefrontal cortex [58].

In the recovery period (15–30 min) following a graded exercise test (GXT), BDNF levels were found to be significantly decreased over those measured at rest [54, 55]. Different from an acute exercise exposure, high intensity aerobic training can increase resting levels of BDNF [61] and promote faster reaction time during an inhibitory control task [62].

The effects of strenuous aerobic exercise on cognition were evaluated in four manuscripts [63–66], two of which were conducted among female military members [65, 66]. The results of these studies consistently identified that exhaustion following aerobic exercise results in poorer cognitive function (i.e., reduced working memory). Additionally, two studies found that cortisol levels increased after a bout of maximal exercise [64, 65]. Evidence indicates that following exhaustive aerobic exercise, working memory assessments decrease by approximately 20% compared to resting values [63]. Similarly, after 3-h of physical exertion, inhibitory control was reduced by 25% when compared to a less intense activity [66].

### Sports Participation and Cognitive-Related Outcomes

Four studies assessed the effects of sports in different conditions: (i) martial arts training session [67]; (ii) comparing open and close skills (soccer and endurance athletes) [68]; (iii) a Padel (racket sport) competition [69]; (iv) skill level of ice hockey players [70]. Results showed that in the recovery period recovery (30 min) following one training session of martial arts [67] and a single Padel competition [69], serum BDNF levels were significantly higher than at rest. Moreover, differences in response were noted between elite and novice hockey players, with elite players showing higher activation of prefrontal and frontal cortex and performing better on executive functions task than their novice peers [70].

Another study compared brain activity and inhibition capacity in participants engaged in open and closed skill sports to those in a control group (lack of specific sports training); sports groups had a better reaction time compared to the control group, independent of the type of sport [68]. However, open-skill sports (e.g., soccer) lead to higher activation in a particular brain region, the basal nuclei (as measured by fMRI) when compared to closed skills (e.g., aerobic athlete) [68].

### Menstrual Cycle Influence on the Relationship Between Cardiorespiratory Fitness, Aerobic Exercise, Sports and Cognitive-Related Outcomes

Regarding reproductive health, two studies evaluated differences between eumenorrheic and amenorrheic (absence of a menstrual cycle) females [71, 72], and another two assessed the impacts of the menstrual phase on cognitive-related outcomes [73, 74].

After maximal aerobic exercise, BDNF (+ 96.5%) and cortisol only increased in the amenorrheic group [71]. At rest, eumenorrheic females showed higher values of BDNF in comparison to those experiencing amenorrhea. Moreover, BDNF positively correlates with estradiol, a significant female reproductive health hormone that is high in the follicular phase triggering events leading to ovulation. [72]

When analyzing inhibitory control, acute aerobic exercise decreased reaction time after exercise, independently of the menstrual phase [73]. BDNF increased after a 20-min bout of vigorous physical activity for both phases (Luteal and follicular) [74]. Conversely, VO_2max_ is negatively correlated with the change in BDNF after a GXT only for follicular phase (r = -0.539) [74].

### Pregnancy Influence on the Relationship Between Cardiorespiratory Fitness, Aerobic Exercise, Sports and Cognitive-Related Outcomes

Three studies investigated the effects of aerobic fitness and exercise on cognitive-related outcomes (biomarkers and cognitive function tests) during pregnancy and after childbirth. Among these 3 studies, one evaluated the impact of an acute bout of submaximal exercise on BDNF [75]; the second study a 16-week intervention that incorporated moderate exercise and BDNF [76]; and the last study investigated the impacts of cardiorespiratory fitness on inhibitory control [77].

After an acute bout of moderate intensity aerobic exercise in pregnant females, serum BDNF increased immediately after exercise. About 10–12 weeks post-delivery, BDNF levels increased and cortisol levels decreased during rest and post-exercise compared to their levels during pregnancy period [75]. Following a 16 week exercise intervention, resting serum BDNF increased (+ 1574.1 pg/ml), while the level of BDNF in the control group, decreased (− 691.9 pg/ml) [76].

Examining inhibitory control responses during pregnancy, Lemoyne and colleagues showed decreased inhibitory control and VO_2max_ across all three trimesters. When VO_2max_ is inserted as a covariate in analyses, it does not change the effect of pregnancy on inhibitory control. These results demonstrate that CRF is not the explanatory variable leading to the decrease in inhibitory control over the course of pregnancy. Also, the non-pregnant control group had a better reaction time and VO_2max_ than the pregnant individuals [77].

## Discussion

### Cardiorespiratory Fitness

The literature surrounding the relationship between CRF and BDNF, found in our scoping review, is inconsistent. While some studies reported an inverse relationship between CRF and BDNF [38, 74], another study found a positive association between these variables [51]. One explanation for the reported inverse relationship could be that BDNF has a fundamental role in tissue repair and formation (vessels, cardiac tissue, bones, skeletal muscles), in this sense circulating BDNF can be mobilized, directed to and taken up by tissues needing repair thereby decreasing circulating levels [34, 78, 79]. The positive associations observed between CRF and BDNF suggest a connection to increased engagement of muscle-type 1 fibers in the context of aerobic activities. It appears that the BDNF-TrkB complex plays a role in fat oxidation processes, crucial for energy generation during aerobic exercise [33, 51, 80], consequently upregulating circulating BDNF levels.

When analyzing the relationship between CRF and executive function, results demonstrated a positive impact of CRF on the activation of the anterior cortex [52]. The executive process depends on brain connections, mainly between the pre-frontal cortex, hippocampus, and basal ganglia [81]. The release of BDNF can be upregulated due to muscular contractions [33] and is consequently linked to CRF. When correlating BDNF with executive functions, it is responsible for synaptic plasticity, long-term potentiation, and long-term memory, promoting higher neuronal activation and improved brain connectivity. This enhancement results in faster processing of tasks [18, 82–84]. Moreover, increased serum BDNF levels are related to a higher hippocampus volume [83]. Taken together these data CRF can increase brain activation and proteins responsible for better cognitive function.

This scoping review found that acute moderate exercise only changes RT among individuals with low fitness levels [52]. Given there is an inverted-U relationship between exercise and cognitive functions, stimulation of the brain could be dependent on the intensity of the bout of exercise [9, 43, 85]. Thus, the cognitive functioning of individuals with higher fitness levels may be less impacted by low/moderate physical activity. In this sense, a higher-fitness individual seems to adapt to metabolic/hormonal changes caused by physical activity and needs more stimulus to promote cognitive gains.

### Aerobic Exercise

Our scoping review focused on females found a positive effect of acute [56] and chronic [60] aerobic exercise on inhibitory control and working memory. The effects of aerobic exercise on executive functions are linked to an increase in neurotransmitters, which can stimulate certain brain areas (e.g., pre-frontal cortex) responsible for cognitive functions [86, 87].

It was determined that there is a positive effect of acute aerobic activity on BDNF. In contrast, during recovery, many studies showed lower serum BDNF than baseline [54–56, 61]. A potential explanation for lower BDNF levels during the recovery period is that muscle damage, which increases BDNF levels in muscle tissue as a necessity for recovery [34, 88], leads to the depletion of stored BDNF in platelets. Moreover, in a rat model, the BDNF is upregulated in soleus after aerobic exercise [34]. Thus, BDNF can bind to TrkB, triggering the repair of damage, increasing muscle regeneration [34, 80], resulting in a decrease in BDNF circulation.

Acute aerobic exercise can lead to an increase in VEGF levels. VEGF is correlated to metabolic demand, with higher exercise efforts leading to higher circulating VEGF [57]. VEGF, stored in muscle fibers, can be secreted during an acute muscle contraction, increasing extracellular levels up to five times resting level. This circulating VEGF stimulates angiogenesis and consequently increases oxygen and metabolite delivery [40]. It is important to highlight the effect of VEGF on angiogenesis within the hippocampus and, consequently, on neurogenesis [41, 89, 90]. In animal model, this increase in VEGF facilitates learning and memory, reducing latency during tasks [89]. The activation of VEGF on brain can also be result from lactate-inducing VEGF. During exercise, lactate levels increase and bind its receptor on the brain (HCAR1). When HCAR1 is activated, it promotes subsequent activation of vascular endothelial growth factor A (VEGFA) and, consequently, brain angiogenesis, mainly in the hippocampus. [91].

A decline in cognitive functions (working memory and inhibitory control) and BDNF levels were seen following exhaustive aerobic exercise. [63, 64, 66]. Moreover, cortisol and the ratio of cortisol to BDNF increases after exertion [64, 65], this increase in cortisol can act as an inhibitor of BDNF synthesis [64, 88]. This finding is important because exhaustive exercise upregulates plasma cortisol levels, increasing catecholamine synthesis, leading to neural noise due to overstimulation of the brain [9, 43]. Cortisol can stimulate the release of glutamate, which binds to NMDA receptors. This interaction can affect synaptic sensitivity and alter BDNF expression, primarily by influencing intracellular calcium influx through NMDA receptors, which can subsequently impact neurogenesis [84, 92, 93]. Moreover, the reduction in BDNF after exhaustive exercise could be related to a shift in the use of additional resources (e.g., lactate for the ATP synthesis) rather than the syntheses of BDNF [45, 94].

Although sex disparities are not the focus of this review, it is important to highlight that studies showed a greater decline in the cognitive function and neurochemical markers of female individuals following exhaustive exercise compared to their male counterparts [65, 66]. Females may be more susceptible to negative sequalae due to disparities in physical fitness and metabolic demands [95]. Consideration should be given to sex disparities when developing training prescriptions.

### Sports Participation

Chronic sports participation can improve cognition, possibly related to high levels of cardiorespiratory fitness that results from the sports participation [26, 29]. But also, sports participation can be independently related to executive functions since it requires higher activation of the prefrontal cortex and higher executive function demand than other forms of physical activity [29].

Of the four studies related to sports participation, one study compared with controls (non-sports participation) [68]. At the same time, other studies analyze the effects of a training/competition session [67, 69] and the impact of skill level on cognition [70]. The results of this review showed positive effects of sports participation on inhibitory control and serum/plasma BDNF. The one study examining different types of sports (open and closed skills), showed the aerobic and soccer groups had faster RT during easier tasks) [68]. The values for RT during an inhibitory task (harder) were faster for the soccer group compared to aerobic and control. A possible explanation for the faster reaction times during inhibitory tasks among the soccer group could be better functional connectivity and activation of certain brain areas (e.g., the basal nuclei and the frontal cortex) [68]. The basal nuclei are responsible for actions such as motor, spatial, visual, and affective. Specifically, the putamen region of the basal nuclei is responsible for motor and visual tasks, being activated during sports, and acting for better inhibitory control [68, 96, 97] and these regions are known to be enhanced during sports, mainly for open skill sports [68].

Moreover, the effects of sports participation, mainly open skills sports, on cognitive control may be related to more complex motor tasks that are required for successful performance in the sport. Open sports require attention and working memory for real-time decision-making, and an increased demand for inhibitory control to ensure corrective action [22, 31, 98, 99].

In essence, there is a lack of comprehensive research concerning how sports impact cognition. This gap stems from the unique cognitive demands of each sport, whether open or closed, and how they contribute to various improvements in aerobic fitness. As a result, the exact enhancements in executive functions linked to sports participation might not have been fully elucidated or might need deeper investigation to consider other influencing factors [29].

### Menstrual Cycle

During the menstrual phase, oscillation in hormonal levels is noted, and estrogen levels are highest between 10 and 14 days of the menstrual cycle [100]. Circulating estradiol can cross blood–brain barriers, and estrogen receptors (ER) are widely distributed in the brain. ER on the membrane can activate signaling pathways responsible for neuroprotection and synaptic formation [101, 102]. Moreover, estradiol can stimulate the brain’s bioenergetic system, improving ATP availability [101]. Estrogen receptors can stimulate the hippocampus, leading to a beneficial effect related to learning, memory, neuronal survival, and neuronal activity [46, 102]. A significant positive association between serum BDNF and estradiol has been noted [72].

It is well known that excessive exercise and weight loss can create an energy deficit that may inhibit the synthesis of gonadal hormones, causing deficits in sexual hormones and menstrual dysfunction [103]. Approximately 25% of runners [103], 15% of ice hockey athletes [104] and 10% of futsal athletes can experience amenorrhea (absence of menstruation) or an irregular menstrual cycle [105]. Among female individuals, reproductive characteristics have been shown to have an effect on cognition. One study suggested that amenorrheic female athletes had lower levels (at rest) of circulating BDNF compared to eumenorrheic female athletes [72]. The presence of BDNF in the endometrium and the discharge associated with menstruation may justify the presences of lower BDNF levels among amenorrheic female individuals. The endometrium may be a source of BDNF synthesis [46, 106] or act as a stimulus for endometrial cell proliferation [107]. Given amenorrheic individuals do not shed their endometrium cyclically (if at all), there is less demand for BDNF, downregulating circulating BDNF levels, with the possibility to decrease the availability of BDNF for the brain once blood BDNF can cross the blood–brain barrier.

Another study found an elevation of BDNF and cortisol levels after aerobic exercise only among amenorrheic athletes [71], possibly suggesting a lack of aerobic stimulus in the eumenorrheic group. Since catecholamines (epinephrine, norepinephrine, dopamine) are regulated by cortisol, and these neurotransmitters/hormones stimulate brain regions [86] regulating metabolite supply [108] this could be an avenue for the increase in BDNF.

This review highlights the effect of acute aerobic exercise on inhibitory control and serum BDNF during both menstrual phases (follicular and luteal), suggesting a beneficial effect of aerobic exercise, independent of menstrual phase [73, 74]. However, this study did not verify the menstrual phase by hormonal dosage [74]. This is a short-coming in the study design, as the presence of menstruation does not equate to normal hormone levels [100].

### Pregnancy

The studies in this scoping review reveal decreased cognitive-related outcomes during pregnancy, characterized by reduced baseline BDNF levels, diminished inhibitory control, and elevated cortisol levels compared to non-pregnant individuals [75, 77]. A possible explanation for these lower levels of BDNF [75] and inhibitory control [77], could be related to higher levels of cortisol during pregnancy [75], with the increase in cortisol down-regulating neurogenesis [92], and affecting neurotransmitters. A single bout of aerobic exercise in pregnancy showed a significant increase immediately after the exercise [75] and exposure to a moderate-intensity aerobic exercise intervention resulted in increased BDNF levels compared to the control group whose levels decreased from baseline [76].

Aerobic exercise can increase BDNF levels during pregnancy [76] and can reduce the deleterious effect of pregnancy on BDNF. Moreover, animal studies have shown higher BDNF levels in offspring from mothers who practiced exercise throughout gestation [76] offering a potential intergeneration benefit. Currently, there is a gap in the literature; investigating the effects of aerobic exercise or CRF on BDNF levels and its receptor in the human placenta, to determine whether there are better metabolic and neurotrophic markers in offspring of mothers who practiced exercise throughout gestation [75]. Maternal adaptations in response to environmental factors (e.g., exercise) can be transmitted to the fetus through the placenta, facilitating the provision of nutrients, hormones, and immunological communications [109, 110]. Finally, BDNF/TRkb can contribute to enhanced fetal growth and may be associated with the management and prevention of fetal growth disturbances [111].

## Limitations

While the aim of this scoping review was to summarize and unveil gaps in the literature related to the benefit of aerobic exercise and sports on cognitive-related outcomes and the possible effect of the menstrual phase or pregnancy on these relationships, it is not without limitations. The review did not assess the quality of the included studies or provide a detailed synthesis of evidence. Additionally, it did not address the heterogeneity among studies. Also, the number of cross-sectional studies limited the interpretation of causality.

## Conclusion

**Fig. 2:**
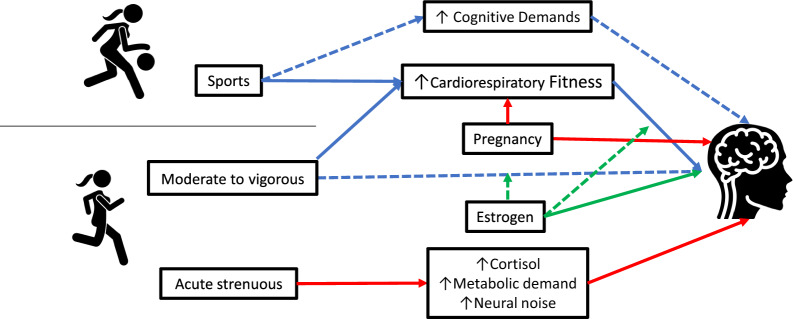
Proposed pathways found in this scoping review between aerobic exercise and sports with cognitive-related outcomes. The continuous line shows consolidated information between variables, whereas dotted lines represent possible pathways that must be elucidated. Blue lines: improvements in outcome; green lines: possible covariates; red lines: deleterious effect on outcome. *Icons used from Flaticon.com

The possible pathways and covariates found is this scoping are elucidated in Fig. 2. The scoping review shows an inverse ‘U’ relationship between the aerobic exercise and cognitive functions, demonstrating an optimal amount and intensity of aerobic exercise to benefit cognitive functions. Also, CRF is significantly related to serum BDNF, but more information is needed to confirm the beneficial effects of CRF on cognitive-related outcomes independent of the menstrual phase since there is a possible effect of estrogen on this relationship. Lastly, no study was found that clearly illustrates the effects of CRF on the BDNF receptor (TrkB) and whether sports practice is independently related to cognitive-related outcomes in females.

### Future Research

Recommendations relating to further research into the roles of cardiorespiratory fitness, aerobic exercise and sports practice in female cognition are presented below:

- Implement precise, objective methods to control for menstrual phases and hormonal variations when analyzing their impact on cognitive performance.
- Investigate the influence of cardiorespiratory fitness on estrogen-cognition dynamics.
- Explore cardiorespiratory fitness as a mediator in sports participation and cognitive-related outcomes.
- Conduct randomized controlled trials to evaluate how aerobic exercise and cardiorespiratory fitness influence cognitive-related outcomes.
- Study the interaction between aerobic exercise/cardiorespiratory fitness and TRKb
- Assess the impact of aerobic exercise/cardiorespiratory fitness on BDNF during pregnancy and in the placenta.

## Data Availability

The datasets generated during and/or analyzed during the current study are available from the corresponding author on reasonable request.

## Abbreviations

ATP: Adenosine triphosphate
BDNF: Brain-derived neurotrophic factor
CRF: Cardiorespiratory fitness
ER: Estrogen receptors
fMRI: Functional magnetic resonance imaging
GXT: Graded exercise test
HCAR1: Hydroxycarboxylic acid receptor 1
NMDA: N-Methyl-D-aspartic acid
PCC: Population, concept, and context
RT: Reaction time
TrkB: Tropomyosin receptor kinase B
VEGF: Vascular endothelial growth factor
VO_2max_: Maximal oxygen consumption
VO_2peak_: Peak oxygen consumption
